# Identification of hot-spot residues in protein-protein interactions by computational docking

**DOI:** 10.1186/1471-2105-9-447

**Published:** 2008-10-21

**Authors:** Solène Grosdidier, Juan Fernández-Recio

**Affiliations:** 1 Life Sciences Department, Barcelona Supercomputing Center, Jordi Girona 29, 08034 Barcelona, Spain

## Abstract

**Background:**

The study of protein-protein interactions is becoming increasingly important for biotechnological and therapeutic reasons. We can define two major areas therein: the structural prediction of protein-protein binding mode, and the identification of the relevant residues for the interaction (so called 'hot-spots'). These hot-spot residues have high interest since they are considered one of the possible ways of disrupting a protein-protein interaction. Unfortunately, large-scale experimental measurement of residue contribution to the binding energy, based on alanine-scanning experiments, is costly and thus data is fairly limited. Recent computational approaches for hot-spot prediction have been reported, but they usually require the structure of the complex.

**Results:**

We have applied here normalized interface propensity (*NIP*) values derived from rigid-body docking with electrostatics and desolvation scoring for the prediction of interaction hot-spots. This parameter identifies hot-spot residues on interacting proteins with predictive rates that are comparable to other existing methods (up to 80% positive predictive value), and the advantage of not requiring any prior structural knowledge of the complex.

**Conclusion:**

The *NIP *values derived from rigid-body docking can reliably identify a number of hot-spot residues whose contribution to the interaction arises from electrostatics and desolvation effects. Our method can propose residues to guide experiments in complexes of biological or therapeutic interest, even in cases with no available 3D structure of the complex.

## Background

Protein-protein interactions are involved in the majority of biological processes and their study from a structural and energetic point of view is increasingly attractive, not only for biological reasons but also for their therapeutic interest [1-3]. Indeed, knowing the binding mode of two interacting proteins, or even better, the residues directly responsible for the interaction (so called 'hot-spots'), could help to the long-awaited goal of disrupting the complex with small molecules [3,4], which would open enormous biological and therapeutic expectations. For this reason, hot-spot residues, typically defined as those residues contributing in more than 1 or 2 kcal.mol^-1 ^to the total binding energy of the complex, are particularly attractive to the pharmaceutical field. Experimental measurement of the residue contributions to binding energy by alanine-scanning is costly, as it requires production by mutagenesis of hundreds of variants that have to be individually analysed by biophysical methods [3,5].

Consequently, the available data on hot-spot residues is quite limited, and many groups have tried to make efficient predictions based on sequence and structural analysis of the known hot-spots.

Protein-protein interfaces are large and characterized by the absence of cavities compared to small-molecule binding sites [6,7]. They are composed of a variety of residues involved in the specificity of the interaction, with a group of quite conserved hot-spot residues acting as binding site anchors that are required in order to stabilize the complex. As the interface gets bigger, the number of hot-spots increases [8]. Hot-spots are surrounded by moderately conserved and energetically less important residues forming a hydrophobic O-ring responsible for bulk solvent exclusion [5,9]. They appear to be clustered in tightly packed regions in the centre of the interface [8]. However, it has not been found any single attribute as shape, charge or hydrophobicity that can unequivocally define a hot-spot by itself [3,6,10,11].

Different scoring schemes for computational hot-spot prediction have been reported, based on residue conservation [12,13], hydrogen bonding [14] or complete energy binding [15-17]. Other approaches have tried a combination of all these parameters with machine learning techniques [18]. Although hot-spot prediction from sequences has been recently reported [10], most of the methods described so far need information from the protein-protein complex structure.

We recently described the normalized interface propensity (*NIP*) parameter [19] obtained from rigid-body docking simulations, which represents the tendency of a given residue to be located at the interface. Here, we will use a variation of this parameter for the prediction of hot-spot residues in a protein-protein interaction without any previous knowledge of the complex structure.

## Results and discussion

### Residue interface propensities from rigid-body docking scored by electrostatics and desolvation

We recently described the residue-based normalized interface propensity (*NIP*) parameter, computed from an ensemble of the 100 lowest-energy ICM ([20]) docking solutions as sorted by a rigid-body docking energy function. The *NIP *values reflected the contribution of every residue to the interface averaged over the lowest-energy docking orientations, and thus could be used to identify surface residues potentially involved in protein-protein interactions [19]. Indeed, a *NIP *cut-off of 0.4 was reported to predict known protein-protein interfaces with positive predicted value (PPV) as high as 81%, but with quite low sensitivity [19]. This low sensitivity for interface prediction would be compatible with the hypothesis that the high *NIP *values are identifying only the very few residues that are really important for the interaction (i.e. the hot-spots). In a similar line of reasoning, a neural network previously developed to identify interface residues, was recently applied for hot-spot prediction from sequences [10]. Thus, the focus of the present work is to explore whether such *NIP *values from docking simulations could be used to predict hot-spot residues in protein-protein interactions. The main advantage is that, in contrast to other current methods, the *NIP*-based predictions could be applied to cases in which no information about the complex structure is available.

In the original *NIP *calculations, a rather time-consuming docking approach was used: ICM-based pseudo-Brownian rigid-body docking search with a complete energy function, including van der Waals, hydrogen bonding, electrostatics and desolvation. Interestingly, we recently showed that electrostatics and desolvation were by far the most important energy terms for rigid-body docking, and they could be successfully used with other faster FFT-based docking methods, as implemented in pyDock [21]. Thus, we have applied here our pyDock approach (fast docking with electrostatics and desolvation scoring) in order to obtain *NIP *values for hot-spot prediction. For that, we first generated alternative docking poses with the known FFT-based docking programs FTDock [22] and ZDOCK [23], which were then scored by pyDock [21] and further analysed with the pyDockNIP module to compute interface propensities (see Materials and Methods). Ideally, for a realistic test, one should use the unbound three dimensional structures of the interacting proteins. In our dataset of 21 cases (Table 1), unbound structures for both ligand and receptor molecules are only available in a few cases (1AHW, 1DFJ, 3HFM, 1JCK, 2PTC).

**Table 1:** Initial dataset of complexes used in this work

Complex^a^	Res^b^	Receptor	Ligand	Unbound receptor	Res^b^	Unbound ligand	Res^b^	Complex type^c^
1A22	2.60	Growth hormone receptor	Growth hormone	-	-	1HGU	2.50	B/U
1A4Y	2.00	Ribonuclease inhibitor	Angiogenin	-	-	1UN3	1.70	B/U
1AHW	3.00	Fab 5G9	Tissue Factor	1K6Q	2.40	2HFT	1.69	U/U
1AIE	1.50	p53	p53	-	-	-	-	B/B
1BRS	2.00	Barnase	Barstar	1A2P	1.50	-	-	U/B
1BXI	2.05	Colicin E9	Immunity protein Im9	1FSJ	1.80	-	-	U/B
1DAN	2.00	Tissue Factor	Factor VII	2HFT	1.69	-	-	U/B
1DFJ	2.30	Ribonuclease A	Ribonuclease inhibitor	1FS3	1.40	2BNH	2.30	U/U
1DN2	2.70	IgG1 Fc fragment	DCAWHLGELV WCT-NH_2_	1H3V	3.10	-	-	U/B
3HFM	3.00	HYHEL-10	HEL	1GPO	1.95	3LZT	0.92	U/U
1GC1	2.50	CD4	gp120	1CDJ	2.50	-	-	U/B
1F47	1.95	Zipa	FTSZ fragment	1F7W	NMR	-	-	U/B
1FC2	2.80	Fc fragment	Protein A	-	-	-	-	B/B
1FCC	3.50	Fc fragment	Protein G	1H3V	3.10	-	-	U/B
1IAR	2.60	IL-4 receptor	IL-4	-	-	1HIK	2.60	B/U
1JCK	3.50	T-cell antigen receptor	SEC3	1BEC	1.70	1CK1	2.60	U/U
1JRH	2.80	Antibody A6	Interferon-γ receptor	-	-	-	-	B/B
1JTD	2.30	TEM-1 β-lactamase	BLIP	1ZG4	1.55	-	-	U/B
1NMB	2.50	NC10	Neuraminidase N9	-	-	7NN9	2.00	B/U
2PTC	1.90	Trypsin	BPTI	1S0Q	1.02	1G6X	0.86	U/U
1VFB	1.80	Antibody D1.3	HEL	-	-	3LZT	0.92	B/U

^a^PDB Code, ^b^Resolution in Å; ^*c*^*B, Bound; U, Unbound*

For most of the complexes, the structure of only one of the two partners is available in the unbound conformation. And for three cases, no unbound structure is known for any of the two partners (1FC2, 1JRH and 1AIE tetramer; Table 1). For instance, in the case of p53 (1AIE), we only have the tetramer conformation, since the monomer is unfolded [24]. In any case, we repeated the docking simulations for the unbound cases but using the bound subunits instead, and did not observe major differences in our results (data not shown). Actually, we always found residues with *NIP *≥ 0.4 except for 3 cases: one unbound/unbound and two bound/unbound (so no clear preferences for bound or unbound can be seen here). In different analysis in the sub-sections below, we will further show that there is no significant difference between using bound or unbound structures. This is somehow expected, as *NIP *is a statistical value averaged over many different low-energy orientations, and thus does not depend so much on the (usually) small bound-unbound differences as it would do if it were based on one single orientation such as the native one. Indeed, we checked that the number of residues with *NIP *≥ 0.4 for a given complex does not depend at all on the existence of near-native orientations in the ensemble of 100 docking poses (data not shown).

### *NIP *value as a predictor for hot-spot residues

**Figure 1:**
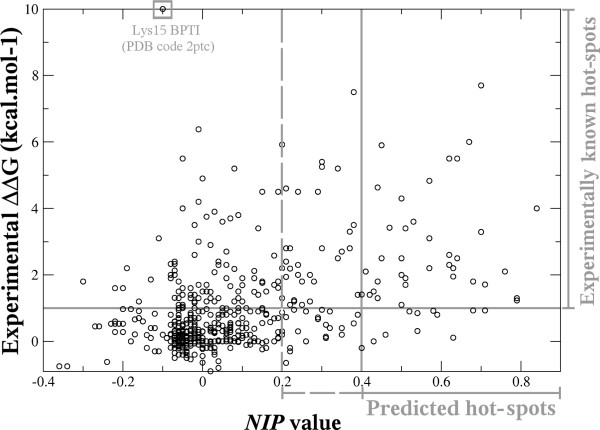
**Experimental binding energy vs. computer predictions**. Distribution of ΔΔG data vs. *NIP *values from rigid-body docking (FTDock+ZDOCK).

Thus, we have analysed whether *NIP *predictions corresponded with hot-spots, by comparing the experimental binding energy (ΔΔG) versus the *NIP *values for the 586 residues with available data in our dataset. As can be seen in Figure 1, we found that most of the predicted hot-spot residues (initially, we consider a prediction when *NIP *≥ 0.4) were indeed experimentally identified as hot-spots (we defined a hot-spot as a residue with ΔΔG ≥ 1.0 kcal.mol^-1 ^upon mutation to alanine, as in Kortemme *et al*. [15]), whereas the majority of the non-predicted residues were correctly identified as non-hot-spots. The overall results are shown in Table 2, and a χ^2 ^test shows that the predictions clearly differ from a random distribution (P < 0.0001). While it is true that many hot-spots are left unidentified (number of false negatives: 128 residues, Table 2; overall sensitivity S= 24%, Table 3), we can still observe high overall statistical significance due to the large proportion of hot-spot predictions that are correct (number of true positives: 40, Table 2; overall PPV = 78%, Table 3) and the number of non-hot-spot residues that are correctly identified (number of true negatives: 407, Table 2). As a matter of fact, the global accuracy of the predictions is 76%. Table 4 details the prediction results for those cases with positive predictions (*NIP *≥ 0.4) also having available experimental data (we have not included here the remaining cases with positive predictions because there is no available experimental data to compare with). These results show that the correct predictions do not just come from a few successful cases. The results did not show any preference for the use of bound or unbound subunits, and all kinds of PPV values can be found in either bound or unbound cases. This is somehow not surprising since in most of the cases the difference between the unbound and bound conformations is around 1 Å RMSD (data not shown). However, we also had good prediction results even in those cases with higher flexibility upon binding (e.g.: 1DN2 with 4.78 Å for unbound/bound receptor RMSD; 1F47 with 2.59 Å for unbound/bound receptor RMSD), which indicates that our method is tolerant to conformational movements. This can be explained because the *NIP *values are computed from the ensemble of docking poses and not from any single orientation, as we previously mentioned. In this line, we could neither found any correlation between the prediction rates and the number and quality of near-native docking solutions (if any) in the docking pools (Table 4). For instance, the 1NMB complex shows the maximum PPV (100%) in spite of not having any near-native conformation (RMSD ≤ 10Å) within the 100 lowest energy docking poses. On the contrary, the 1JRH complex shows one of the smallest PPV values (50%), in spite of having as many as 10 near-native conformations in the docking ensemble. All these results reinforce the fact that *NIP *values come from some general features of the low-energy docking ensemble and not from any single conformation in particular. These docking ensembles are driven by our pyDock docking energy, favouring docking orientations where most contributing residues (i.e. hot-spots) can form similar interactions to those in the native state (even though these docking orientations do not necessarily correspond with near-native binding modes). In this way, our predicted hot-spots are likely to be those residues that have favourable desolvation upon binding independently on the partner interaction region (i.e. apolar and aromatic residues), but not those exposed residues involved in specific hydrogen bonding or electrostatics interactions, especially when become buried upon binding (in which case, our method cannot precisely describe the favourable interactions and thus cannot compensate the high desolvation penalty). Thus, our method predicts 52% of the Tyr and 46% of the Phe hot-spot residues in our database. However, it predicts only 7% of the Arg hot-spots, in spite of being one of the most abundant residue types in hot-spots [9]. Other polar hot-spots (Glu, Lys) are also poorly predicted (8% and 0%, respectively).

**Table 2:** Comparison between *NIP *predictions (cut-off 0.4) from rigid-body docking and the experimentally known hot-spot residues

	*NIP *≥ 0.4	*NIP *< 0.4	Total
ΔΔG ≥ 1^a^	40	128	168
ΔΔG < 1^a^	11	407	418
Total	51	535	586

^a ^in kcal.mol^-1^

**Table 3:** Benchmarking *NIP *hot-spot predictions on different datasets

	***PPV***	***S***
Initial dataset (21 cases)^*a*^		

NIP ≥ 0.2	68%	43%
NIP ≥ 0.4	78%	24%
FOLDEF	73%	46%
ROBETTA^*b*^	71%	69%

Li's dataset (15 cases)^*c*^		

NIP ≥ 0.2	75%	42%
NIP ≥ 0.4	91%	26%
FOLDEF^*d*^	70%	45%
ROBETTA^*d*^	64%	60%

Additional dataset (all 22 cases)^*e*^		

NIP ≥ 0.2	59%	34%
NIP ≥ 0.4	78%	15%
Additional dataset (X-ray subunits)^*e*^		

NIP ≥ 0.2	73%	44%
NIP ≥ 0.4	80%	19%

Additional dataset (NMR subunits)^*e*^		

NIP ≥ 0.2	0%	0%
NIP ≥ 0.4	0%	0%

Additional dataset (modeled subunits)^*e*^		

NIP ≥ 0.2	59%	33%
NIP ≥ 0.4	75%	15%

Comparison to ROBETTA and FOLDEF

^a ^Our initial dataset (Table 1); ^b ^Values from Kortemme and Baker,^14 ^on a sub-set of 19 complexes from our initial dataset; ^c ^Dataset compiled by Li et al.;^28 d ^Values from Li et al.;^28 e ^Our additional dataset (Table 6).

**Table 4:** Detailed results for hot-spot predicted residues (NIP ≥ 0.4) with available experimental data.

Complex	Number of predicted residues(*NIP *≥ 0.4)	hot-spot prediction success(PPV)	Number of near-native poses^a^
1BRS	5	100%	25
1BXI	6	67%	6
1DFJ	3	100%	8
1DN2	1	100%	5
1F47	3	67%	1
1JCK	7	100%	0^b^
1JRH	2	50%	10
1NMB	1	100%	0^c^
1VFB	9	67%	1
3HFM	3	100%	0^d^
1IAR	7	71%	1
1AIE	4	50%	3

^*a *^Number of near-native solutions (RMSD ≤ 10 Å) within the ensemble of 100 lowest-energy docking orientations used to calculate the NIP values; ^*b*^best RMSD within the ensemble is 22.0 Å; ^*c*^best RMSD is 20.9 Å; ^*d*^best RMSD is 12.2 Å.

Given that perhaps the most important contribution to our docking energy is the desolvation term [21], we have also analysed whether desolvation alone (which can be calculated on the individual molecules without actually performing the docking simulations) could be a good predictor for hot-spots. However, we found no correlation at all between the known hot-spots and the ASA-based desolvation calculated either per individual residue or as in the ODA method [25] (data not shown). As it happened for rigid-body docking results [21], it seems that the optimal hot-spot prediction comes from the combination of desolvation and electrostatics energy terms. Additional terms, such as van der Waals, do not actually improve the hot-spot prediction results (data not shown). This is probably due to the fact that docking poses generated by FTDock and ZDOCK have already some level of shape-complementarity (within the extent of the rigid-body approach), so only desolvation and electrostatics are required to describe the optimal docking ensemble.

On the other side, given that our *NIP *calculations are applied to all protein surface residues without applying any restraints, a number of hot-spot predictions might incorrectly appear away from the interface and thus contribute to the false positive rate. However, when we only consider interface residues (i.e. that have at least one atom at <5Å from any atom of the partner molecule in the known complex), our predictions (*NIP *≥ 0.4) do not significantly change (data not shown). This further shows that our method does not need extra experimental information about the native interface or the complex structure.

### *NIP *threshold for optimal predictions

**Figure 2:**
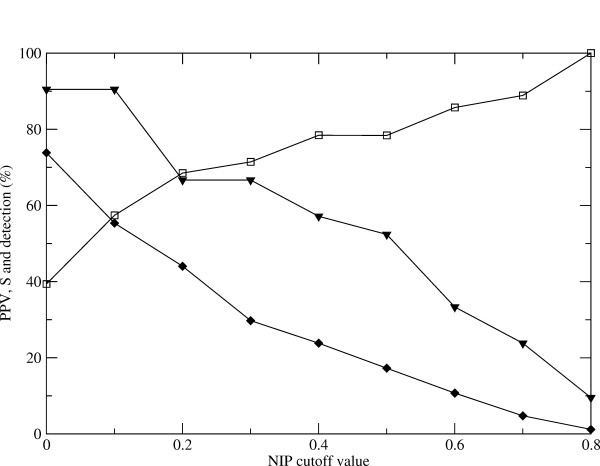
**Global performance of hot-spot prediction**. Evaluation of prediction results according to the *NIP *cut-off value: global PPV (open squares), global sensitivity (diamonds), and percentage of cases with prediction (triangles).

Using the original cut-off of *NIP *≥ 0.4 yields quite reliable predictions, but this somehow limits the number of predicted residues, and consequently, the sensitivity value can never be too high. Different cut-off values have been tested in order to find the optimal predictive rates (Figure 2).

Interestingly, as the *NIP *cut-off value increases, the PPV systematically improves, although the sensitivity decreases as expected. On the other side, as the *NIP *cut-off value gets lower, the number of cases with hot-spot prediction expectedly increases. Thus, a *NIP *cut-off of 0.2 could give a good compromise between PPV and sensitivity. The overall results of these predictions (*NIP *≥ 0.2) are shown in Table 5, and a χ^2 ^test shows that the predictions clearly differ from a random distribution (P < 0.0001). With this *NIP *cut-off of 0.2, the PPV decreases slightly to 68%, but the sensitivity improves up to 43%, while the global accuracy also improves to 78% (Table 3). In summary, as a general rule, one could base the hot-spot prediction on any of these two cut-off values, depending on the aim of the work. If the goal is to identify a significant number of possible hot-spots, a reasonable option is to use the general cut-off of *NIP *≥ 0.2. On the other side, if the aim is to detect a few residues with optimal reliability, for instance in order to guide site-directed mutagenesis experiments, it would be much more sensible to use the stricter criteria of *NIP *≥ 0.4.

**Table 5:** Comparison between *NIP *predictions (cut-off 0.2) from rigid-body docking and the experimentally known hot-spot residues

	NIP ≥ 0.2	NIP < 0.2	Total
ΔΔG ≥ 1^*a*^	73	95	168
ΔΔG < 1^*a*^	34	384	418
Total	107	479	586

^a ^in kcal.mol^-1^

### Comparison to other hot-spot prediction methods

A recently reported method, ROBETTA [15], showed a PPV of 71% and a sensitivity of 69% on a set of 19 cases (a sub-set of our 21 cases) for the prediction of hot-spots (with ΔΔG ≥ 1.0 kcal.mol^-1 ^upon mutation to alanine), as can be seen in Table 3. Our method (*NIP *≥ 0.2) gives worse sensitivity (S = 43%) than ROBETTA, while the PPV is similar (PPV = 68%). A stricter cut-off of *NIP *≥ 0.4 yields much better PPV (78%), although fewer residues are predicted. The main advantage of our method is that it does not require the atomic 3D structure of the complex while ROBETTA does.

Another reported method to compute *in silico *alanine-scanning on protein-protein complexes is FOLDEF [26]. Since in their original publication they do not give hot-spot predictive rates nor detailed data to calculate them, we have run this program through a server ([27]) in order to compare the results. On our 21-complexes dataset (Table 1), FOLDEF yielded a PPV of 73%, with a sensitivity of 46% for the prediction of hot-spots (ΔΔG ≥ 1.0 kcal.mol^-1 ^upon mutation to alanine). These values are comparable to the ones obtained with our predictions using *NIP *cut-off ≥ 0.2 (Table 3). Again, the results are encouraging as our method does not need the complex structure while FOLDEF does.

A more recent benchmark has been reported on a set of 15 complexes [28], in which ROBETTA had slightly worse predictive rates (PPV = 64%; S = 60%) for the prediction of hot-spots (with ΔΔG ≥ 1.0 kcal.mol^-1^) than in the larger set of 19 cases, while FOLDEF gave similar results (PPV = 70%; S = 45%) to the ones in the larger set of 21 complexes. Our predictions (*NIP *≥ 0.2) gave a PPV of 75% and a sensitivity of 42%, more in line with FOLDEF (Table 3). Interestingly, with stricter cut-off (*NIP *≥ 0.4) our method obtained an excellent PPV (91%) at the expense of sensitivity.

Thus, our method is comparable to ROBETTA and FOLDEF, with the advantage that ours does not require the structure of the complex. To the best of our knowledge, the only other hot-spot prediction method that does not require the complex structure is actually based on sequence analysis alone [10]. They reported a reasonable performance (PPV = 60%; S = 66%), although the true positives were defined as those predicted residues with experimental ΔΔG > 2.5 kcal.mol^-1 ^whereas the false positives were defined as those predicted residues with experimental ΔΔG = 0 (thus the "true" PPV, i.e. when using ΔΔG < 2.5 to define false positives, will be probably lower than that reported in that work).

We could compare our results with interface prediction methods, many of which can be applied on the individual proteins without performing docking. Although they have not been specifically developed for hot-spot prediction, we could still evaluate their performance for the sake of comparison. As an example, our ODA algorithm [25] is one of the most successful binding site prediction methods, and is precisely using the same desolvation energy as pyDock. We have already mentioned that prediction of hot-spots with our ODA method is worse than that of the *NIP*-based predictions. For instance, when we use the recommended cut-off for interface prediction (ODA = -10), we obtain a good PPV of 75% but a quite low sensitivity of 7%. It seems that the ODA method is identifying a few hot-spot residues with highly favourable desolvation upon binding but is clearly missing other residues that can be better identified by the *NIP*s from the docking ensembles. We have also analysed one other well-known binding site prediction method, ProMate [29]. We used the ProMate server ([30]) according to the default parameters, and we considered as predicted interface residues as those with the 10% of highest scores, but only if the score was above 0.7 (as their authors suggested in the publication). We evaluated the performance as hot-spot predictor in our dataset of 21 cases, and the results were also clearly worse than those of the *NIP *predictions (PPV = 49%; S = 19%).

The standard binding site prediction methods identify residues involved in the interaction, independently on whether they are hot-spots or not (as expected, given that these methods were not developed to focus on the most energetically contributing residues). However the *NIP *values, derived from energy-based docking ensembles, are able to detect the residues that are energetically important for the interaction. Perhaps it would be interesting to combine several of the binding site prediction methods in order to better identify hot-spots, however this is beyond the present study.

### Successful hot-spot predictions

**Figure 3:**
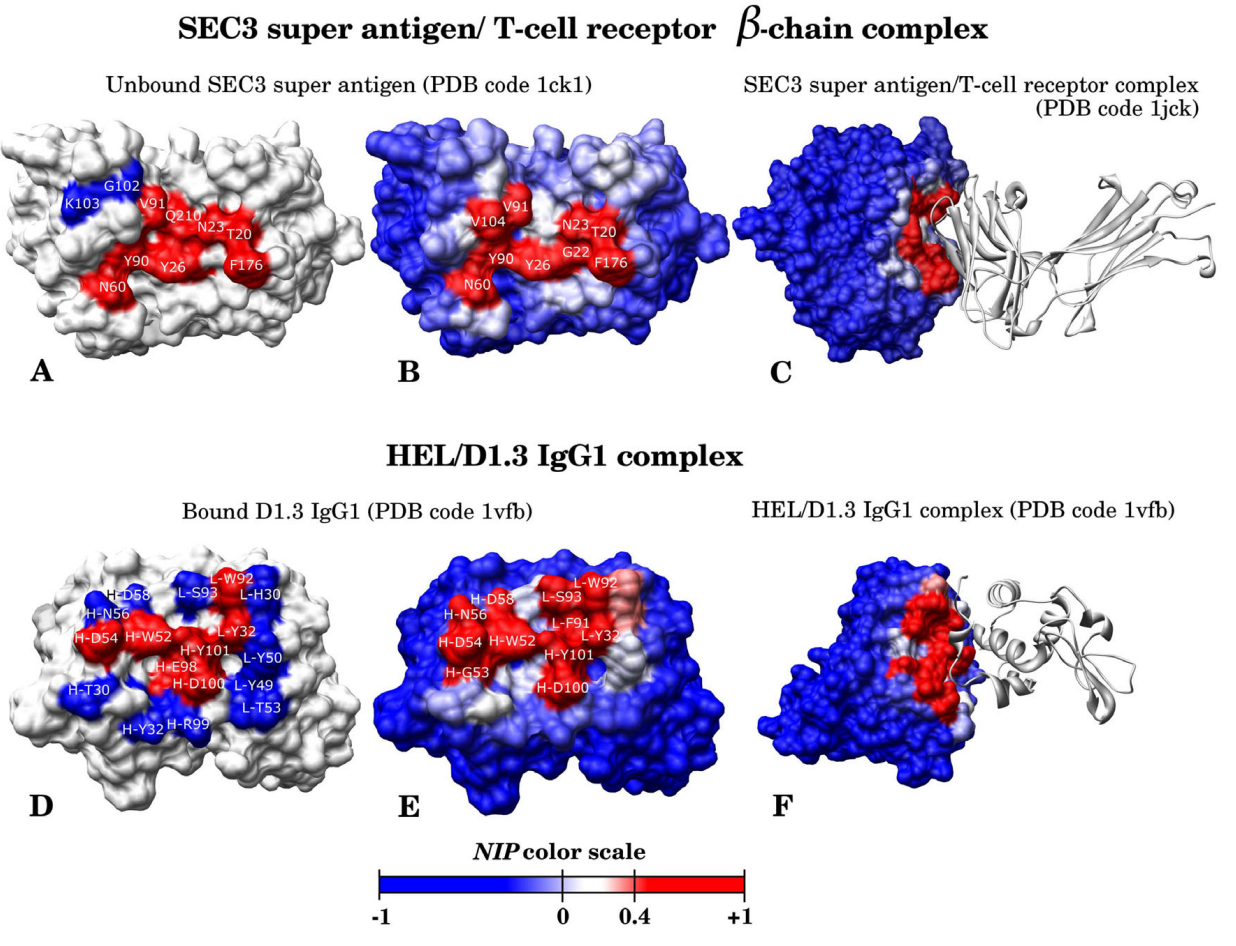
**Examples of hot-spot predictions**. Selected complex examples: SEC3 super antigen/T-cell β-chain (A to C) and HEL/D1.3 IgG1 (D to F). (A, D) Experimental data: hot-spots in red (ΔΔG ≥ 1 kcal.mol^-1^); non hot-spots in blue (ΔΔG < 1 kcal.mol^-1^). Only residues with available experimental data have been shown with labels. L- and H- in IgG1 labels indicate residues belonging to the light and heavy antibody chain respectively. (B, E) Computer predictions. Residues predicted to be hot-spots (*NIP *≥ 0.4) are shown in red; the remaining residues are shown in a scale from red to blue. Only residues with *NIP *≥ 0.4 have been labelled. (C, F). Complex X-ray structures (PDB codes 1JCK and 1VFB, respectively). Receptor residues are coloured according to the *NIP *values. Ligand is represented as a grey ribbon.

We report here two examples, corresponding to the SEC3 super antigen in complex with T-cell receptor β-chain (complex PDB 1JCK), and D1.3 IgG1 in complex with HEL (complex PDB 1VFB). The two binding sites have long been well studied, and plenty of experimental data concerning both hot-spots and non-hot-spots are available, which make these cases particularly interesting for the evaluation of our predictions. The method is able to find correctly the crystallographic interface in both cases (Figure 3), as expected from our previous study [19]. Comparing the experimental hot-spot data (Figure 3A) with the predictions (*NIP *≥ 0.4; Figure 3B) for the SEC3 super antigen, we correctly predicted seven residues as hot-spots (N23, T20, Y26, N60, Y90 -T90 in the complex-, V91 and F176 -P176 in the complex-), two residues as non-hot-spots (G102 and K103 -L103 in the complex-), while only one hot-spot was incorrectly predicted as non hot-spot (Q210). This corresponds to a PPV of 100% and a sensitivity of 87.5%. Concerning the D1.3 antibody (Figure 3E), 13 residues were correctly predicted (six as hot-spots: L-Y32, L-W92, H-W52, H-D54, H-D100, H-Y101 and seven as non-hot-spots: L-H30, L-Y49, L-Y50, L-T53, H-T30, H-Y32, H-R99) whereas four residues were badly predicted (one hot-spot incorrectly predicted as non not-spot: H-E98 and three non-hot-spots incorrectly predicted as hot-spots: L-S93, H-N56, H-D58), which corresponds to a PPV of 66.6% and a sensitivity of 85.7%. The *NIP *values are thus able to predict hot-spots with a global accuracy of 90% for the SEC3 super antigen, and 76.5% for the D1.3 Igg1. Interestingly, we predicted as hot-spots two residues in the SEC3 super antigen (V104 and G22) and another two in D1.3 IgG1 (L-F91 and H-G53) for which there is no available experimental data. Although valine, glycine and phenylalanine are not especially abundant residues in hot-spots [9], according to our predictions they could be interesting for future alanine-scanning experiments.

### Cases with incorrect predictions in our dataset

Most of the false negative hot-spots come from a few particular complexes. For instance, the method (with *NIP *≥ 0.4) was not able to find any of the 18 known hot-spots for the Interferon (IFN)-γ Receptor in complex with the AntiBA6 antibody. The formation of this complex is believed to be more complicated than a simple rigid-body docking in a lock-and-key-like process. Indeed, the overall backbone structure of IFN-γ Receptor in complex with AntiBA6 antibody (complex PDB 1JRH) differs from that of the same receptor in complex with its natural ligand IFN-γ (PDB 1FYH) on the CC' surface loop [31]. However, we used here the coordinates of IFN-γ Receptor and AntiBA6 as directly taken from the reference complex (PDB 1JRH) since unbound structures are not available, so conformational change upon binding does not seem to explain the poor predictions in this case.

The most dramatic false negative, with ΔΔG = 10 kcal.mol^-1 ^(Figure 1), corresponds to the BPTI residue K15 in the BPTI-Trypsin complex (complex PDB 2PTC). Actually, the unbound BPTI (PDB 1G6X) that we used in the simulations has precisely this residue mutated to Arg, which could (at least partially) explain the highly incorrect prediction. However, we found other significant false negatives in different cases that cannot be explained by the existence of a mutation in the unbound molecule. Many of these residues are involved in highly specific hydrogen bonding or electrostatics interactions with the partner molecule. Actually, interactions with very specific geometric requirements are less likely to be predicted by our method. As we have discussed in previous sub-sections, a residue with high *NIP *value is typically involved in many different docking poses with good binding energy, independently on the orientations that these docking poses may have. Thus our method is more likely to predict apolar and aromatic hot-spot residues that have favourable desolvation upon binding independently on the partner interaction region, and thus can be reproduced in other orientations in a similar way to that of the native state. On the other side, it will be more difficult to predict hydrogen bonding or electrostatics interactions from exposed residues that are buried upon binding. The binding contribution of these residues arises from a fine balance between a favourable hydrogen bond or electrostatics interaction and the strong desolvation penalty of burying charged atoms. But these specific favourable interactions are difficult to describe in other conformations different from the native one, so the global contribution will be underestimated by our method. In accordance to this, we have seen above that our method predicts quite well the Tyr and Phe hot-spot residues, but very badly the Lys, Arg, and Glu hot-spot residues.

In several cases, our method predicted residues that were located far from the considered complex interface, in regions that were actually involved in binding to other proteins. For example, in the complex between Tissue Factor and Fab 5G9, the residue F50 was predicted as hot-spot for Tissue Factor but it was situated exactly to the other side of the protein-Ab interface. Interestingly, we found that this residue was located in the interface of Tissue Factor in complex with Factor VII (PDB code 1DAN), although it is not described as hot-spot (ΔΔG = 0.4 kcal.mol^-1^).

In the case of SEC3 super antigen in complex with T-cell receptor β-chain, the residues A102, Q106, F107, F108 were predicted as hot-spots for T-cell receptor, but instead of being located in the interface of interest, they were actually situated in the interface between the α and β chains (PDB 1D9K). A few predictions for immunoglobulin Fc fragments in their CH2-CH3 domains were identical between the complexes 1FCC and 1FC2 (with Protein G and Protein A respectively). Interestingly R301, which has been predicted as hot-spot in both complexes, is implicated in the interaction of Fc fragment with the Fcγ Receptor, as proved by the fact that R301A immunoglobulin mutant shows a decrease in binding to several Fcγ Receptor types [32].

As a final comment, experimental alanine-scanning mutagenesis results must be taken with caution. Indeed, if a given mutation introduces a dramatic conformation change in the unbound interacting protein affecting the overall complex stability, then a ΔΔG variation above 1 kcal.mol^-1 ^could be observed, even if the residue involved is not directly participating in the interaction with the partner [3]. These cases should not be strictly considered as hot-spots for binding, and in any case, they would be impossible to predict by our method.

### Additional benchmarking on an extended dataset

We also benchmarked the docking and hot-spot predictions on an additional dataset (Table 6), different from our initial test set (see Materials and Methods), and where the 3D structure of the complex is not available for most of the cases. When using a *NIP *cut-off of 0.4, the overall predictions on this additional dataset yielded a PPV of 78%, similar to that of the initial data set (Table 3).

**Table 6:** Additional dataset of complexes used in this work

Complex^*a*^	Res^*b*^	Receptor	Ligand	Receptor PDB	Res (Id)^*b*^	Ligand PDB	Res(Id)^*b*^	Complex type^*c*^
X-ray subunits								

1CBW	2.60	Chymotrypsin	BPTI	4CHA	1.68	4PTI	1.50	U/U
1PM9	1.70	IL-6R	IL-6	1N26	2.40	1ALU	1.90	U/U
N/A	N/A	KDR	VEGF	2P2H	1.95	1VFP	2.50	U/U
N/A	N/A	trkC	Neurotrophin-3	1WWC	1.90	1NT3	2.40	U/U
2BTF	2.55	Rabbit actin	Bovine profilin I	1J6Z	1.54	1PNE	2.00	U/U
1DVF	1.90	E5.2	D1.3	-	-	1VFA	1.80	B/U
N/A	N/A	sHIR	Insulin	2DTG	3.80	2C8Q	1.95	U/U

NMR subunits								

N/A	N/A	GPIIbIIIa	Kistrin	1TYE	2.90	1N4Y	NMR	U/U
N/A	N/A	bFGF	FGFR1b	4FGF	1.60	1WVZ	NMR	U/U
N/A	N/A	IGF-1R	IGF-1	1IGR	2.60	2GF1	NMR	U/U
N/A	N/A	IGF-1bp	IGF-1	1ZT3	1.80	2GF1	NMR	U/U

Homology-based modeled subunits								

N/A	N/A	E9 DNase	Im2	1FSJ	1.80	1FR2^*d*^	(66%)	U/M
N/A	N/A	AChR	Erabutoxin	2BG9	4.00	5EBX	2.00	cryo- EM/U
N/A	N/A	AChR	NmmI	2BG9	4.00	1V6P	0.87	cryo- EM/U
1Z92	2.80	IL-2 receptor	IL-2 (human)	2B5I^*d*^	(100%)	1M47	1.99	M/U
N/A	N/A	IL-2 beta receptor(human)	IL-2 (murine)	2B5I^*d*^	(99%)	1M48^*d*^	(64%)	M/M
N/A	N/A	IL-2 alpha receptor(murine)	IL-2 (murine)	1NWV^*d*^	(22%)	1M48^*d*^	(64%)	M/M
N/A	N/A	IL-4/IL-4bp	GammaC	1IAR	2.30	2B5I^*d*^	(99%)	U/M
N/A	N/A	gp75	Neurotrophin-3	1LNL^*d*^	(18%)	1NT3	2.40	M/U
N/A	N/A	CD48	CD2	2DRU^*d*^	(39%)	1CDC	2.00	M/U
N/A	N/A	Calcineurin	CaM	1AUI^*d*^	(59%)	1LKJ	NMR	M/U
2D9Q	2.80	hG-CSFbp	hG-CSF	2D9Q^*d*^	(99%)	1RHG	2.20	M/U

^a ^PDB Code; N/A, Not Available; ^b ^Resolution in Å or (Id): sequence identity with template in case of model; ^c ^B, Bound; U, Unbound; M, Model; cryo-EM, cryoelectron microscopy structures classified as modeled structures because of the low resolution compared to crystallographic or NMR structures, ^d ^PDB code of the template structure used for modelling

However, only a few of the 94 known hot-spots were detected, so the corresponding sensitivity (*S *= 15%) was lower than that of the initial dataset (*S *= 24%). When *NIP *≥ 0.2, the overall predictions gave a PPV of 59% and a sensitivity of 34%, slightly worse values than those of the initial dataset (Table 3).

This additional dataset was more heterogeneous than the initial one, in the sense that the coordinates of the unbound subunits were in some cases taken from X-ray structures, in other cases from NMR models, and in some other cases they needed to be modelled based on homology (see Materials and Methods). In order to minimize the effect of the input structures quality on the predictions, we classified this dataset on three different groups and analysed the predictive results accordingly (Table 3). On the one side, when we considered only the cases in which we used the crystallographic structure of the interacting subunits, we had an overall PPV of 73% and a sensitivity of 44% (with *NIP *≥ 0.2; Table 3), very similar to the results obtained in the initial dataset (when using *NIP *≥ 0.4, the results were also similar to those of the initial dataset). It is interesting that in this sub-set all cases are unbound-unbound (except for one bound/unbound), while in the initial dataset there were five unbound/unbound, 13 bound/unbound and three bound/bound cases. Again, the fact that we obtained very similar results in both datasets shows that the predictions do not depend on whether we used the bound or unbound subunits, as discussed before. On the other side, when we considered only the cases with modelled subunits, the overall PPV was 59% and the sensitivity 33% (*NIP *≥ 0.2), values that were slightly worse than those of the initial dataset. This is somehow expected, as modelled structures will have more uncertainty than unbound X-ray structures. Finally, when we considered only the cases with unbound NMR structures, the results were strikingly poor, with a PPV of 0% and a sensitivity of 0%, either with *NIP *≥ 0.2 or *NIP *≥ 0.4. For some reason our method is not working correctly on NMR structures. For the molecule IGF-1, we used the minimized average NMR structure (PDB 2GF1). As for the rest of NMR structures, there were not minimized average structures provided in the PDB, so we used instead the first model of the NMR ensemble. A similar behaviour of our desolvation energy on NMR structures was already previously reported [25].

When using the stricter *NIP *cut-off of 0.4, we obtained predictions for the following four cases with experimental data: D1.3/E5.2 (docking unbound/bound), Im2/E9 DNase (docking model/unbound), Erabutoxin/AChR (docking model/unbound) and the murine complex IL-2/IL-2 alpha receptor (docking model/model). Concerning the D1.3/E5.2 complex, a total of 8 hot-spots were correctly detected on D1.3 on both light and heavy chains but there were no predictions for the E5.2 antibody. Regarding the Im2/E9DNase interaction, the model shared 66% of sequence identity with its template and permitted correct predictions of three hot-spots, with two of them displaying a ΔΔG variation above 5.8 kcal.mol^-1^. As for the Erabutoxin/AChR complex, we considered the AChR subunit as a model given that the structure comes from low-resolution cryo-electron microscopy experiments. Most of the predicted residues had actually high experimental ΔΔG variation residues; indeed, among the 12 hot-spots with a ΔΔG above 4 kcal.mol^-1^, five of them were correctly predicted. Regarding the IL-2/IL-2 alpha receptor complex, although sequence identities with the templates were only of 68% and 22% respectively, our method predicted successfully the strongest hot-spot of the interaction. What is interesting is that our predictions seem to work almost equally well when using homology-based modelled structures than with X-ray structures. Moreover, the good predictive results in this additional set, in which most of the cases do not have available the 3D structure of the complex, proves the capabilities of our method and opens the possibility of large-scale hot-spot predictions.

## Conclusion

We present here the application of docking simulations to predict hot-spots for protein-protein interaction without prior knowledge of the complex structure. The *NIP *values, computed from docking ensembles as scored by electrostatics and desolvation, can be used to identify with high reliability (around 80% positive predictive value) a number of hot-spots that are directly contributing to the interaction, due to electrostatics and water-to-interface desolvation effects. On the down side, the method is not exhaustive and cannot predict all possible hot-spots in an interaction, especially those that are not directly involved in the interface or that arise from the formation of highly specific interactions. In summary, our method can propose residues to guide mutational experiments in complexes of biological and therapeutic interest, even if the 3D structure of the complex is not available.

## Methods

### Dataset description

We used two different hot-spot datasets for our analysis. The first one is derived from 21 complexes with available 3D structure that have already been used in previous hot-spot prediction studies [14,26]. This dataset (Table 1) includes enzymes-ligand/inhibitor complexes (PDB code: 1JTD, 1BRS, 1BXI, 1A4Y, 1DFJ, 2DAN, 2PTC), antibody-antigen complexes (PDB code: 1DN2, 1FCC, 3HFM, 1FC2, 1NMB, 1AHW, 1VFB, 1JRH, 1JCK) and other types of interaction (PDB code: 1IAR, 1AIE, 1F47, 1CG1, 1A22). This set has a total number of 8168 surface residues, from which 888 are interface residues. Among them, there have been described 168 hot-spot residues (ΔΔG ≥ 1 kcal.mol^-1 ^when mutated to alanine) and 418 non-hot-spot residues (ΔΔG < 1 kcal.mol^-1^), as can be seen in Table 2. All the available mutational data can be found on the Alanine Scanning Energetics database ([33], Thorn and Bogan [34]) or in already published hot-spots studies for 1DN2, 1NMB, 1JRH, 1F47, 1FCC [14], 1JTD and 1AIE [26].

We compiled later a second dataset (Table 6), in order to test our method on a higher number of cases, which is composed of 22 additional complexes found on the Alanine Scanning Energetics database (most of them without available 3D complex structure), and includes 94 hot-spot and 267 non-hot-spot residues. It comprises enzyme/inhibitor complexes (BPTI/chymotryspin, Im2/E9DNase), toxin/receptor complexes (Erabutoxin/AChR, Nmmi/nAChR), cytokine/receptor complexes (IL-6/IL-6R, IL2 (human)/IL-2 receptor, IL-2 receptor beta (human)/IL-2 (murine), IL-2 (murine)/IL-2 alpha receptor (murine), IL-4/IL-4bp/Gamma C, h-CSF/hG-CSFbp), growth factor/receptor or binding protein complexes (bFGF/FGFR1b, VEGF/KDR, IGF-1/IGF-1R, IGF-1/IGF-1bp, BMP type IA receptor/BMP-4), and other complexes (bovin profilinI/rabbit actin, kistrin/GPIIbIIa, D1.3/E5.2, sHIR/Insulin, CD2/CD48, CaM/calcineurin, neurotrophin-3/gp75, neurotrophin-3/trkC). In the cases with no experimental structure for any of the unbound subunits, we used for docking the homology-based models generated from MODBASE, when available ([35], Pieper *et al*. [36]). In order to avoid redundancy, we have removed all the numerous cases involving hGH bound to different antibodies.

### Prediction of hot-spots from docking simulations

We have explored the use of a variation of our previously described Normalized Interface Propensity (*NIP*) values [19] derived from rigid-body docking simulations, for the prediction of hot-spots. The rigid-body docking procedure is divided in two steps. The sampling of the ligand around the receptor was first achieved using two known FFT-based programs: FTDock 2.0 [22], which generates 10.000 different solutions (or docking poses), and ZDOCK 2.1 [23], which gives 2000 solutions. All docking poses from both programs were then ranked together, based on an optimized scoring function formed by electrostatics and desolvation energy, as implemented in the PyDock algorithm [21]. The 100 lowest-energy solutions (this number of solutions was previously shown to be adequate for computing the NIP values) [19] were next selected to be analysed by pyDockNIP, which is an implementation of a previously described method able to predict interface propensities from the distribution of docking poses [19].

The Averaged Buried Surface (ABS_i_) for each residue was calculated from the 100 lowest-energy solutions:

$$ABS_{i}=\frac{1}{N}\sum_{k=1}^{N}\left(\frac{ASA_{i}^{Unb}-ASA_{ik}^{Bnd}}{ASA_{i}^{Unb}}\right)$$

where $ASA_{i}^{Unb}$ is the solvent-accessible surface area for the receptor residue i before ligand binding, $ASA_{ik}^{Bnd}$ is the solvent-accessible surface area for the same residue after ligand binding according to the docking pose k.

These ABS values were normalized in order to obtain a Normalized Interface Propensity:

$$NIP_{i}=\frac{ABS_{i}-\langle ABS\rangle }{ABS^{MAX}-\langle ABS\rangle }$$

where ⟨*ABS*⟩ is the average ABS value, and *ABS*^*MAX *^the maximum expected ABS value (*ABS*^*MAX *^= 1).

A Normalized Interface Propensity (*NIP*) value of 1 would indicate that the corresponding residue is involved in all predicted interfaces of the 100 lowest energy docking solutions whilst a value of 0 would mean that it appears as expected from random. A negative value would mean that the residue appears less often than expected from random. A threshold of 0.4 was tested previously and found to be the best compromise between detection and positive predictive value (PPV) for binding site prediction using a different dataset [19]. Accordingly, in this article we have considered as hot-spot predictions those residues with *NIP *values higher or equal to 0.4.

### Statistical significance of hot-spot prediction

In order to analyse whether the residues predicted as hot-spots differ significantly from a random prediction, a χ^2 ^test was performed. The distribution of predicted hot-spots for all complexes was compared with that of the known hot-spot residues. A 2 × 2 contingency table was built by computing the number of predicted residues (*NIP *≥ 0.4 or *NIP *≥ 0.2) that are experimentally known hot-spots and the ones that are not hot-spots, as well as the number of not predicted residues (*NIP *< 0.4 or *NIP *< 0.2) that are known hot-spots and the ones that are not hot-spots. The *P *significance obtained by applying this χ^2 ^test represents the probability to obtain these predictions just by chance. The lower the value of *P*, the higher our confidence that the predictions are significantly different from random.

### Comparison to other methods for hot-spot prediction

To compare our results with the ones than can be obtained using different existing methods, we used the dataset composed of 15 different complexes (1A4Y, 1AHW, 1BRS, 1BXI, 1CBW, 1DAN, 1DFJ, 1DVF, 1FC2, 1GC1, 1JCK, 1VFB, 2PTC, 3HFM, 3HHR) from the work of Li *et al*. [28] We compared our method with ROBETTA [15] and FOLDEF [26] for 294 residues (N293A and W45A from 3HHR and 1DAN respectively, originally in Li's dataset, were removed due to mutations in unbound structures used for docking with pyDock). Unlike our method, the 3D structure of the complex is a prerequisite for ROBETTA and FOLDEF, which consist in complete energy binding calculations. Additionally, the FOLDEF server (version 1.10) was used to compute all the ΔΔG values of our dataset (Table 1).

### Assessment of predictions: sensitivity and positive predictive value

To analyse further the relevance of our predictions, the statistical parameters of positive predictive value (PPV), sensitivity (S) and global accuracy have been computed.

$$PPV=\frac{TP}{TP+FP}$$

$$S=\frac{TP}{TP+FN}$$

$$GlobalAccuracy=\frac{TP+TN}{TP+FP+FN+TN}$$

with *TP *the number of True Positives, *FP *the number of False Positives, *TN *the number of True Negatives and *FN *the number of False Negatives.

The positive predictive value (PPV) was defined as the fraction of predicted residues that were correctly described as hot-spots, and the sensitivity (S) was the fraction of real hot-spot residues that were actually predicted.

## Abbreviations

*NIP*: normalized interface propensity; ASA: accessible solvent area; ASP: atomic solvation parameters; ODA: optimal docking area; PPV: positive predictive value; S: sensitivity; SEC3: staphylococcal enterotoxin C3; HEL: hen egg lysozyme; IFN-γ: interferon-γ; BPTI: bovine pancreatic trypsin inhibitor; Im2: immunity protein 2; AchR: acetylcholine receptor.

## Authors' contributions

JFR devised the concept and directed the research. SG performed the calculations. SG and JFR analysed the data. SG drafted the paper and JFR finalized the draft. All authors read and approved the final manuscript.
